# A macro-level analysis of the socio-economic impacts of climate change driven water scarcity: Incorporating behavioural and resilience aspects

**DOI:** 10.1016/j.wroa.2024.100223

**Published:** 2024-04-16

**Authors:** Andrew G Ross, Kevin Connolly, Stefan Vögele, Wilhelm Kuckshinrichs

**Affiliations:** a Institute of Energy and Climate Research (IEK), Systems Analysis and Technology Evaluation (IEK-STE), Forschungszentrum, Jülich, Germany; b Fraser of Allander Institute, Department of Economics, University of Strathclyde, Glasgow, Scotland, United Kingdom

**Keywords:** Water shortage, Water scarcity, Socio-economic impacts, Impact assessment, Behavioural keynesian, Resilience, Ecosystem services, Natural capital

## Abstract

Recognising the urgent need to address water scarcity resulting from climate change, there is a growing push to enhance the resilience of water (and related) systems. For instance, policymakers are now urging companies to shift from short-term focused strategies towards long-term approaches to effectively manage water scarcity. This paper utilises a custom-built dynamic multisectoral model to assess the socio-economic impacts at a macro-level of temporary water scarcity. The focus of the analysis is to identify the effects that varying levels of investment foresight may have on economic resilience. Specifically, the model incorporates often overlooked factors such as behavioural and resilience aspects. By considering these key elements, a more comprehensive understanding of the system-wide implications of water scarcity on the broader economy is provided. The analysis shows how firms' foresight, or lack thereof, impacts their response to water scarcity and the subsequent impact on the economy. Sector-specific analyses shed light on the potential negative impacts of water scarcity on sectors like agriculture, food, and electricity production and distribution. Yet, the analysis also reveals that certain sectors can benefit from competitiveness effects, which can mitigate the adverse economic implications of water scarcity. However, it should be noted that these sectors may contribute to a catch-up effect on water use. The policy recommendations arising from this research emphasise the promotion of anticipation and preparedness among firms. It is crucial to prioritise resilience-building measures in all sectors, whether they directly rely on water or not.

## Highlights

• Model water scarcity along with macro-level behavioural and resilience aspects.
• Assess how investment decisions influence the macro-economy during water scarcity.
• Proactive investment planning is essential to mitigate climate change impacts.
• Industry must be incentivised to invest in measures that reduce vulnerability.

## Introduction

Climate change will exacerbate water stress and water scarcity (IPCC, 2023). Water scarcity refers to periods of reduced water availability, often caused by factors such as insufficient rainfall or drought. On the other hand, water stress occurs when there is high consumption of water relative to its availability (Kummu et al., 2016). The consequences of water scarcity are particularly detrimental to economic sectors that heavily rely on water. Agriculture, for instance, suffers from lower crop yields due to insufficient soil moisture. Furthermore, lowered river discharges not only affect electricity production in thermal, nuclear, and hydropower plants but can also impact inland shipping by reducing water depth in rivers (EEA, 2021). These consequences, in turn, affect all other sectors of the economy.

Given the expected increase in frequency and intensity of water scarcity, the importance of resilience in water systems and governance is gaining recognition. This is especially crucial considering ongoing climate change and global environmental uncertainties. However, in the current literature assessing the impacts of these events, the importance of resilience is often overlooked (Rodina, 2019).^1^

This paper specifically focuses on resilience in relation to the economy's capacity to recover after a disturbance, with a particular emphasis on the decision-making strategies employed by firms in response to water scarcity.^2^ Many studies in this field use a myopic model specification (this is outlined in more detail in Supplementary Material S1), which is justified given the challenges of assessing unpredictable and discontinuous extreme events using foresight models. Nonetheless, it is crucial to acknowledge the value of foresight models despite their limitations, particularly in light of current efforts to encourage firms to incorporate long-term planning (EuropeanCommission, 2021). These models can provide valuable insights into the benefits of proactive decision-making, especially in scenarios where immediate benefits may not be realised. With foresight, it is possible to avoid stranded investments resulting from decisions made under myopic planning. Despite the recognition of the importance of employing models with various investment specifications in fields such as energy transition modelling (Hanna and Gross, 2021; McCollum et al., 2020), this approach has not yet been embraced in research specifically focused on water (scarcity).

The primary aim of this paper is to investigate how different investment behaviours, incorporating resilience behavioural characteristics, impact the macro-economy during and after periods of temporary water scarcity. To accomplish this, three distinct investment model specifications are utilised: perfect foresight, myopic expectations, and imperfect foresight. The perfect foresight model assumes that firms can accurately predict and plan for future water scarcity. Conversely, the myopic expectations model imposes that firms only adjust their capital stocks based on current prices and outputs, potentially leaving them unprepared for future challenges. Finally, the imperfect foresight model posits that firms predict future output based on past trends, which in turn may lead to inaccurate predictions.

Two scenarios are simulated to identify the system-wide resilience to water scarcity. In the first scenario, there is a single month-long water scarcity event in May. In the second scenario, both May and August experience continuous water scarcity throughout the entire month, with the August event being more severe. These scenarios are further specified in the simulation scenario section. Subsequently, the three investment behaviour model specifications are simulated, resulting in a total of six sets of simulation results.

This paper contributes to policy and literature by examining economic resilience and its implications for decision-making strategies by investigating the macro-level socio-economic impacts of water scarcity. By examining different investment decision models, the analysis highlights the significance of taking proactive measures and quantifies the varying outcomes of investment strategies at the individual sector level. These findings have direct relevance for climate change adaptation, offering guidance to policymakers in developing strategies that incentivise industries to invest in measures that mitigate climate change impacts.

## Results and discussion

In this section, the analysis utilises the DEMACRO-ESS model, a general equilibrium (CGE) model specifically focused on Germany.^3^ This custom-built model incorporates ecosystem services (ESS), primarily water and land, and adopts a modelling approach based on standard economic theory. This approach allows for causal interpretations of the model's numerical results. The DEMACRO-ESS model serves as a numerical tool to facilitate analytical reasoning and enables the examination of how the economy responds to changes in specific parameters or external shocks. It offers a comprehensive understanding of system dynamics by isolating the effects of specific variables while holding all others constant. This allows for a thorough examination of the economy's responses (Ross et al., 2024).

One of the key advantages of this modelling approach is its ability to not only identify qualitative effects but also quantify the potential magnitudes of effects across different economic variables (Dixon and Jorgenson, 2012). The detailed modelling utilised provides insights into potential impacts at the sectoral level, which is a crucial aspect of the analysis. Moreover, the DEMACRO-ESS model addresses often neglected aspects of economic resilience by integrating different investment behaviour specifications and incorporating an imperfectly competitive labour market closure that allows for wage bargaining behaviour.

**Fig. 1:**
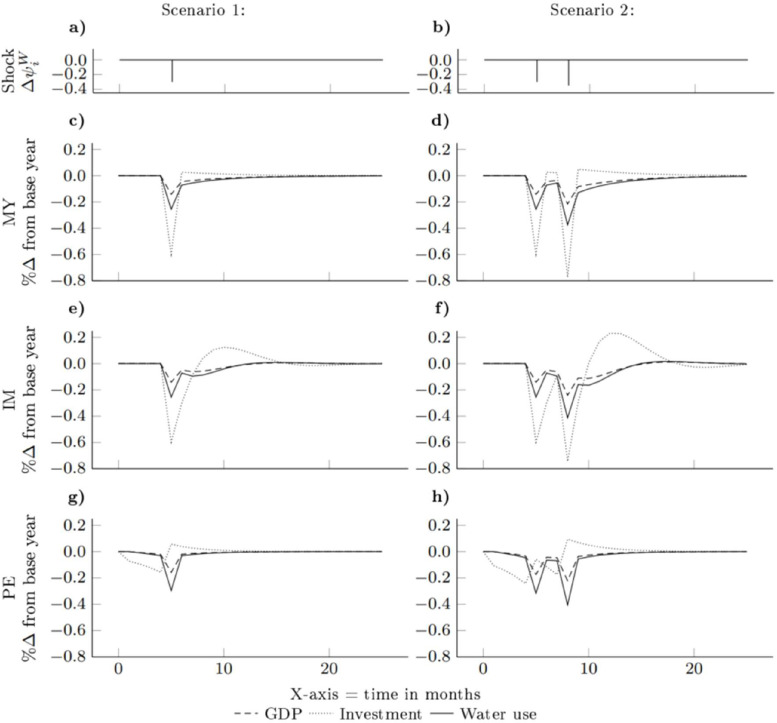
Comparison of aggregate transition paths of GDP, investment, and water use during temporal water scarcity events for Scenarios 1 and 2: DEMACRO-ESS simulation results with different investment behaviour model specifications. MY = Myopic; IM = Imperfect foresight; PE = Perfect foresight; GDP = gross domestic product. Values in percentage changes from base year.

The time path adjustments for the key variables, gross domestic product (GDP), investment, and water use, during a temporal water scarcity event using the three investment model specifications (MY = Myopic; IM = Imperfect foresight; PE = Perfect foresight) are presented in Fig. 1. Each simulation period represents one month. The results for the single water scarcity event in May are depicted in Figs. 1a, c, e, and g, while Figs. 1b, d, f, and h showcase the results for the concurring water scarcity events in May and August.^4^ Note that employment effects are not depicted on this graph for tractability, as they closely track the path of GDP. Additionally, Table 1 provides a summary of the main effects on GDP, investment, employment, and water use during temporal water scarcity events for Scenarios 1 and 2. The results in this table include information on selected time periods and present the cumulative impacts by summing across all time periods.

**Table 1:** Impacts on GDP, investment, employment, and water use during temporal water scarcity events for Scenarios 1 and 2. The results provide information on selected time periods and present the cumulative impacts by summing across all time periods. DEMACRO-ESS simulation results with different investment behaviour model specifications. Values in percentage changes from base year.

		Scenario 1 Selected time periods			Total (all time periods)	Scenario 2 Selected time periods			Total (all time periods)
		5	8	10		5	8	10	
GDP	MY	−0.14	−0.03	−0.02	−0.38	−0.14	−0.22	−0.07	−0.89
	IM	−0.14	−0.06	−0.03	−0.39	−0.14	−0.24	−0.11	−0.89
	PE	−0.16	−0.01	−0.01	−0.27	−0.17	−0.22	−0.03	−0.68
Investment	MY	−0.60	0.02	0.01	−0.45	−0.60	−0.77	0.04	−1.04
	IM	−0.60	0.04	0.12	−0.45	−0.60	−0.74	0.00	−1.04
	PE	0.06	0.02	0.01	−0.25	−0.06	0.09	0.05	−0.68
Employment	MY	−0.17	−0.03	−0.02	−0.37	−0.17	−0.25	−0.06	−0.86
	IM	−0.17	−0.05	−0.02	−0.37	−0.17	−0.27	−0.10	−0.86
	PE	−0.17	−0.01	0.00	−0.27	−0.18	−0.23	−0.02	−0.66
Water use	MY	−0.26	−0.04	−0.03	−0.60	−0.26	−0.37	−0.10	−1.39
	IM	−0.26	−0.09	−0.04	−0.60	−0.26	−0.41	−0.17	−1.38
	PE	−0.30	−0.01	−0.01	−0.46	−0.32	−0.40	−0.04	−1.13

**Note:** MY = Myopic; IM = Imperfect foresight; PE = Perfect foresight; GDP = gross domestic product.

In response to water scarcity shocks, various general effects can be observed. The decrease in the availability of water as an input factor causes a decline in production and subsequent reduction in aggregate employment. Workers, faced with rising unemployment, find themselves with diminished bargaining power and, as a result, suffer wage cuts. The decline in demand consequently leads to lower aggregate prices. Nevertheless, this initiates competitiveness effects, ultimately resulting in an increase in exports that partially mitigate the negative consequences of water scarcity. Despite this, these mitigating effects are inadequate to completely counteract the repercussions of the water scarcity, leading to an overall decline in GDP. After the water scarcity period(s), the economy undergoes a gradual adjustment back to its initial state, driven by investments. These general effects align with previous literature and economic theory (Bekchanov et al., 2017; Wittwer, 2019). However, the quantitative effects and adjustment paths are heavily influenced by investment behaviour, and individual sectors may experience distinct effects. This is not explicitly identified/modelled in the existing literature (further details can be found in Supplementary Note S1). The subsequent text provides a comprehensive overview of the overall results derived from the model specification that incorporates myopic expectations. Following this, a brief discussion of the results for the alternative investment decision approaches is provided. Additionally, a discussion of the sector-specific outcomes is included to provide a more nuanced understanding of the impacts.

In the myopic expectations model, firms are not prepared for water scarcity and do not take any preventive measures. This leads to a decrease of 0.14% in GDP^5^ and a 0.17% decline in employment (see Table 1 data column 1) during the drought period in scenario one. It takes approximately ten time periods for GDP to recover, as shown in Fig. 1b, driven by an increase in investment. In scenario two, a more severe shock occurs after the initial one, and once again, firms do not anticipate it. This scarcity leads to a decline of 0.22% in GDP and a 0.25% decrease in employment during the second drought period as shown in Table 1 data column 6. By time period 25, all variables have adjusted to the shock in this scenario. Overall, in scenario one, there is a 0.38% fall in GDP, a 0.37% fall in employment, a 0.45% fall in investment, and a reduction in water use of 0.6%. In scenario two, these effects amount to a 0.89% fall in GDP, a 0.86% fall in employment, a 1.04% fall in investment, and a 1.39% reduction in water use.

In the imperfect-foresight case, firms with heuristic investment behaviour experience similar initial effects to the myopic case, with a reduction in GDP and employment. However, the recovery is slower after the shock, and average GDP is slightly lower than with myopic expectations. Firms in the imperfect foresight model overestimate previous periods' output, leading to an over-investment that impacts GDP. It takes several periods for the model to stabilise, with some minimal catch-up effects on GDP in later time periods. The negative impacts are prolonged in the second scenario. It is noteworthy that the overshoot in investment in the imperfect foresight specification equalises the negative effects across all simulation periods compared to those seen in the myopic case, despite the overall slow adjustment.

In the model with perfect foresight investment specification, firms anticipate the upcoming shock and adjust, resulting in a slight reduction in economic activity beforehand. In the first scenario, there is a reduction in GDP before the drought occurs, followed by a further reduction during the drought, though the impact is sharp. However, the recovery to initial levels is rapid compared to the myopic and imperfect foresight models. Similar effects are seen in the second scenario, with a sharp impact and a rapid recovery.^6^

**Fig. 2:**
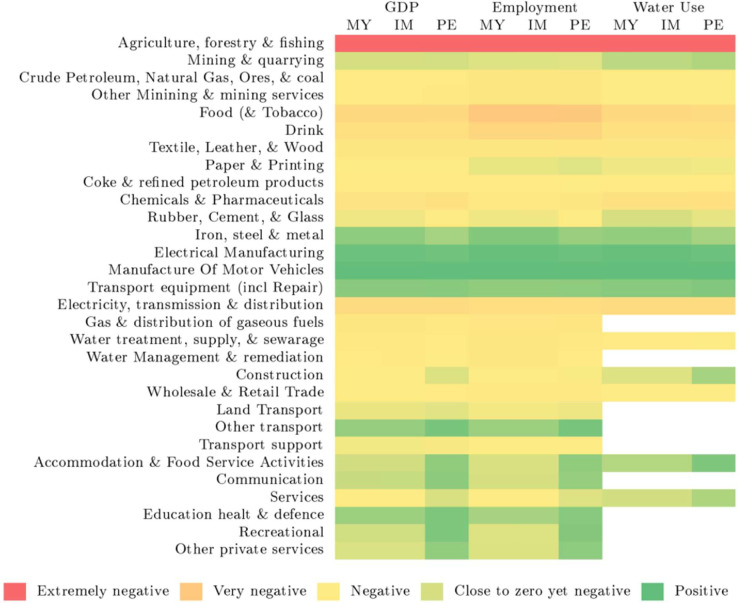
Impact of different investment behaviour models on sector effects of GDP, investment, and water use during temporal water scarcity events (Scenarios 1 and 2): DEMACRO-ESS simulation results. Colour legend: red - extremely negative effects, orange & yellow - less negative effects, light green - close to zero yet negative, green - positive results. MY = Myopic; IM = Imperfect foresight; PE = Perfect foresight; GDP = gross domestic product. The corresponding quantitative results are given in Appendices B and C.

Fig. 2 provides an overview of qualitative sectoral effects for GDP, employment, and water use across all simulation periods for the three investment behaviour specifications for the two scenarios. The corresponding quantitative results are given in Appendices B and C. The qualitative findings depicted in Fig. 2 remain consistent across the two scenarios, despite variations in the magnitude of effects – hence the reporting in one comprehensive figure.

From these results, several key observations can be made. Firstly, numerous sectors are consistently negatively impacted across all scenarios and investment model specifications. The agriculture sector (denoted in red) experiences significant adverse effects, as expected, due to its direct dependence on water availability. The food, and the electricity production and distribution sectors (in orange) are also substantially affected by the assumed drought conditions in the two scenarios. These industries use freshwater in their processes and are directly impacted by water scarcity. On the other hand, there are some sectors that do not follow the overall trend. These sectors primarily benefit from competitiveness effects, where falling prices and resulting trade effects mitigate the contractionary impacts. This positive outcome (highlighted in green) is particularly noticeable in the motor vehicle sector and its related activities. However, these sectors also contribute to a catching-up effect in terms of water use in the periods following the water scarcity.^7^ The divergences in results between the myopic and imperfect foresight model specifications, as observed at the aggregate level, are not significantly pronounced at the individual sector level. However, when comparing these two model specifications to the perfect foresight model, more noticeable distinctions emerge (as also evident in the aggregate results). In this case, investment foresight plays a crucial role in mitigating the extent of negative effects arising from the water scarcity events.

The main results of the analysis can be summarised as follows. In the myopic expectations model, firms lack anticipation of water scarcity. This results in a decline in GDP and employment during the drought periods. However, over time, there is a gradual recovery of GDP. While the initial effects are negative, the economy demonstrates some resilience in slowly rebounding. Similarly, in the imperfect foresight model, firms still fail to anticipate the water scarcity, leading to similar initial effects as in the myopic case. However, the recovery is slower, indicating reduced resilience. The absence of foresight prolongs the period of economic decline, impeding a swift recovery. In contrast, the perfect foresight model shows that firms anticipate the water scarcity and there is a slight decrease in economic activity before the drought occurs. While the water scarcity does have a significant impact, the economy rapidly recovers to initial levels. This indicates a high level of resilience to water scarcity when foresight is incorporated into decision-making processes. These patterns are further emphasised when examining individual sectors within the economy. The agriculture sector, due to its direct dependence on water availability, experiences substantial adverse effects in all scenarios. Additionally, the food and the electricity sectors are also significantly affected. However, certain sectors, such as the motor vehicles and related activities, exhibit positive effects due to competitiveness (trade) effects, resulting in increased output and employment. Nonetheless, these sectors contribute to a catching-up effect in terms of water usage.

## Conclusions

The focus of the analysis is to identify the effects that varying levels of investment foresight may have on economic resilience, incorporating often overlooked factors such as behavioural aspects. By considering these key elements, a comprehensive understanding of the system-wide implications of water scarcity on the broader economy is provided. The analysis highlights the impact of firms' foresight or lack thereof on their response to water scarcity, as well as the subsequent effects on the economy. Sector-specific analyses shed light on the potential negative impacts of water scarcity. Additionally, the analysis reveals that certain sectors might benefit from competitiveness effects, which can cushion the adverse economic implications of water scarcity, although this may contribute to increased water use via catch-up effects. Importantly, the analysis presents both qualitative findings and quantifies the potential magnitude of these effects. To improve the analysis, it would be valuable to extend it to a multi-country context, especially considering the potential competitiveness effects. Given the current policy efforts to address the insufficient investment by industries in measures to protect against and mitigate the impacts of climate change, the findings of this analysis carry significant importance. Policymakers should prioritise promoting anticipation and preparedness among firms, as those with some level of foresight perform better during water scarcity events. It is crucial for these activities to cover all sectors, regardless of their direct reliance on water. Additionally, establishing policies that prioritise enhancing resilience, especially in water-dependent sectors, is recommended.

## Materials and methods

The analysis presented in this paper is based on a custom built dynamic multi-sectoral CGE model, called DEMACRO-ESS. The model is based on the AMOS CGE modelling framework (Lecca et al., 2013; McGregor et al., 2021) and incorporates (neo) Keynesian characteristics. A full list of equations of the base models is given in Lecca et al. (2013) and Ross et al. (2023). The DEMACRO-ESS model extends the DEMACRO model (Ross et al., 2023, 2024) by considering in detail eco-systems services, specifically water and land; and by implementing aspects of resilience via the investment foresight closures. The model is parameterised on a 2020 Social Accounting Matrix (SAM) for Germany which is based on the EXOBASE Input-Output tables (Stadler et al., 2018) and other publicly available data following the approach outlined in Emonts-Holley et al. (2014). The SAM is at monthly frequency such that each simulation time period represents a month.

As mentioned earlier, the adopted modelling approach has several strengths. It is based on standard economic theory, allowing for causal interpretations of numerical results, and serves as a useful numerical tool to explore potential future events and government policies. The model framework enables controlled experimentation and sensitivity analysis, offering a comprehensive understanding of system dynamics (refer to Appendix A for detailed sensitivity analysis). However, there are also limitations to the approach. It relies on numerous parameters that may be challenging to quantify, implying that the presented results should be interpreted as scenarios rather than precise predictions (Ross et al., 2024).

The DEMACRO-ESS model has three domestic transactors: households, corporations, and government; four major components of final demand: consumption, investment, government expenditure, and exports; the industrial sectors; and one type of labour. Land and water are ecosystem-services within capital. Real government expenditure is exogenous and remains fixed (in terms of specific physical quantities). The demand for German exports is determined via conventional export demand functions and imports are obtained through an Armington (1969) link with trade substitution elasticities of 2.7 (Bajzik et al., 2020). Financial flows are not explicitly modelled, with Germany assumed to be a price-taker in financial markets. Tax rates are fixed, and government expenditure are held constant in real terms.

**Fig. 3:**
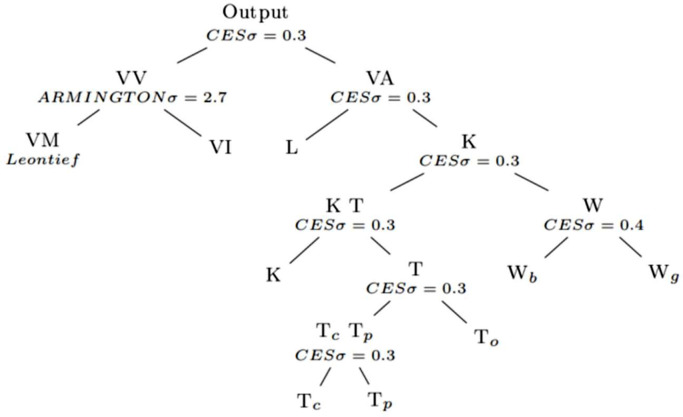
DEMARCO-ESS production structure. CES = Constant elasticity of substitution; VV = Intermediates; VM = Domestic intermediates; VI = Imported intermediates; VA = Value-added; K = Capital; *T* = Land (*c* = crops; *p* = pastures; *o* = other); *W* = Water (*g* = green; *b* = blue).

Production takes place in perfectly competitive industries using multi-level production functions, as illustrated in Fig. 3. This implies that in every time period all commodity markets clear with price equal to the marginal cost of production (Lecca et al., 2013). The model, however, allows for imperfections in the labour market, generating involuntary unemployment. Value-added (VA) is produced using capital (K), which is broken down into land (T) and water (W) and labour (L) - the introduction of land and water into the modelling is outlined in the next section. Intermediates (VV) are broken down by domestic (VM) and imported (VI). In each industry intermediate purchases are modelled as the demand for a composite commodity with fixed (Leontief) coefficients. These are substitutable for imported commodities via an Armington (1969) link. In general, constant elasticity of substitution (CES) technology is adopted with substitution elasticities equal to 0.3 (Mućk, 2017), so that input substitution occurs in response to changes in the relevant relative factor-prices.

### Land and water

The standard production function of the DEMACRO model (Ross et al., 2023) is extended in DEMACRO-ESS to include water and land as natural capital. This is accomplished through a nested process, as illustrated in Fig. 3. Value added is divided into labour and capital. Initially, the natural capital is separated from capital and then further divided into water and land. The model incorporates two types of water - green (W_g_) and blue (W_b_) - and three types of land capital - crops (T_c_), pastures (T_p_), and other (T_o_). Capital is determined by a CES relationship between other capital and water:

(1) $$D_{i}^{K}=\psi_{i}^{TW}{\left[\alpha_{i}^{K}D{{}_{i}^{T}}^{\rho }+\beta_{i}^{K}D{{}_{i}^{W}}^{\rho }\right]}^{\frac{1}{\rho }}$$

where $D_{i}^{K}$ is the demand for total capital in sector *i*, $\alpha_{i}^{K}$ and $\beta_{i}^{K}\;$are the shape and share parameters for the total capital nest based on the information given in the SAM. $D_{i}^{T}$ and $D_{i}^{W}$ are the demands for other capital (land and physical) and water (blue and green) with $\psi_{i}^{TW}$ and being the respective factor efficiency, and ρa parameter of substitution. Note that in the modelling analysis, the scenarios are incorporated by reducing the water efficiency parameter, $\psi_{i}^{W},$ in Eq. (1) for the respective months.

### Labour market behaviour

In all simulations the labour force is fixed, but employment is variable over time, the unemployment rate can change, and labour is mobile across sectors. A bargained real wage (BRW) function, essentially a wage curve (Blanchflower and Oswald, 1995), is employed, which allows for involuntary unemployment, to reflect behavioural aspects within the aggregate labour market. This is a positive empirical relationship between the real consumption wage and workers bargaining power, which is inversely related to the unemployment rate so that:

(2) $$\ln\left(rw_{t}\right)=\gamma -\epsilon \ln\left(un_{t}\right)$$

where $rw_{t}$ is the after-tax real wage at time *t*, un is the unemployment rate (set initially to 4%), ϵ is the unemployment rate elasticity which is set to 0.1 (Longhi et al., 2006), and γ is a calibrated parameter so as to replicate base year data. The paper does not consider the effects of migration. However, the modelling framework employed in this study is capable of conducting a thorough analysis of migration and its impacts (McGregor et al., 2021).

### Investment behaviour

In the perfect foresight closure, the optimal time path of investment is derived following Hayashi (1982) by maximising the present value of the firms’ cash flow, subject to a capital accumulation function, $K_{i,t}$, so that:

(3) $$Max\;\sum_{t=0}^{\infty }\left(\frac{1}{1+r}\right)\left[\pi_{t}-I_{t}\left(1+g\left(w_{i,t}\right)\right)\right]$$

subject to: $K_{i,t+1}=K_{i,t\;}\left(1-\delta \right)+I_{i,t}$where, r is the interest rate, $\pi_{t}$, is the firm's profit, $I_{i,t}$, is private investment, $g(w_{i,t})$ is the adjustment cost function with $w_{i,t}=I_{i,t}/K_{i,t}$, and δ is depreciation rate.

In the myopic closure, gross investment is equal to depreciation, δ, plus some proportion τ, of the difference between the desired capital stock in the next time period, $K_{i,t+1}^{*}$, and the actual capital stock, $K_{i,t}$, so that:

(4) $$I_{i,t}=\tau \left[K_{i,t+1}^{*}-K_{i,t}\right]+\delta K_{i,t}$$

The desired capital stock in period t+1 is determined by the output price, p, and cost of capital, r, in time period t, and the expected output in the following period, $Q_{i,t+1}^{e}$ so that:

(5) $$K_{i,t+1}^{*}=K_{i}\left(Q_{i,t}^{e}\;p_{i,t}\;r_{i,t}\right)$$

The firm therefore takes existing industry output as the best estimate of output in the next period, $Q_{i,t+1}^{e}=Q_{i,t}$. (See Lecca et al. (2013) for a detailed discussion of the myopic and the perfect-foresight closures within the AMOS modelling framework).

In the imperfect foresight version firms are again forward looking but instead of expectations of a fully solved general equilibrium based on Eq. (3), a more simple heuristic approach is assumed (Allan et al., 2020). That is firms base their expected future output on a linear extension of previous output of n periods such that:

(6) $$Q_{i,t+1}^{e}=Q_{i,t}+\frac{Q_{i,t}-Q_{i,t-n}}{n}=\frac{\left(n+1\right)Q_{i,t}-Q_{i,t-n}}{n}$$

Similar to the myopic specification the model is solved based on (4), (5) but the expected output is based on the linear extrapolation of previous output based on 6.

### Simulation scenario

To assess the economic resilience to water scarcity, two fictitious scenarios are assumed. In the first scenario, a month-long water scarcity event is assumed, lasting the entirety of May in a given year. In the second scenario, concurring water scarcity events are assumed to occur in both May (repeating the first scenario) and August (also lasting the entire month), with the latter being more severe. This closely aligns with the historical (and expected) experience of drought events in Germany (Glaser and Kahle, 2020; Ionita et al., 2021; Petrovic et al., 2022; Rakovec et al., 2022; Toreti et al., 2019). In the modelling analysis, these scenarios are incorporated by introducing reduction in water efficiency, $\psi_{i}^{W},$ in Eq. (1) for the respective months – an approach commonly employed (Bekchanov et al., 2017; Calzadilla et al., 2016). Specifically, in the first scenario, a 30% decrease in water efficiency during the month of May is assumed. Similarly, in the second scenario, water efficiency is again reduced by the same percentage in May and by 35% in August. These simulations are carried out using the three investment behaviour models, resulting in a total of six comprehensive sets of simulation results.

## Funding

This work was supported in part by the Helmholtz Association under the project “Helmholtz platform for the design of robust energy systems and their supply chains” (RESUR). Open Access is funded by the Deutsche Forschungsgemeinschaft (DFG, German Research Foundation) (491111487).

## Declaration

During the preparation of this work the authors used OpenAI to increase readability. After using this tool, the author reviewed and edited the content as needed and take full responsibility for the content of the publication.

## CRediT authorship contribution statement

**Andrew G Ross:** Conceptualization, Data curation, Formal analysis, Funding acquisition, Investigation, Methodology, Validation, Visualization, Writing – original draft, Writing – review & editing. **Kevin Connolly:** Conceptualization, Data curation, Formal analysis, Investigation, Methodology, Validation, Writing – original draft, Writing – review & editing. **Stefan Vögele:** Writing – review & editing. **Wilhelm Kuckshinrichs:** Conceptualization, Writing – review & editing.

## Declaration of competing interest

The authors declare that they have no known competing financial interests or personal relationships that could have appeared to influence the work reported in this paper.

## Figures and Tables

**Table A1:** Impacts on GDP, investment, employment, and water use during temporal water scarcity events for Scenarios 1 and 2 with varying openness of the economy. The results give the cumulative percentage change impacts by summing across all time periods. DEMACRO-ESS simulation results with different investment behaviour model specifications. Values in percentage changes from base year.

		Scenario 1- total impacts			Scenario 2- total impacts		
		(all-time periods)			(all-time periods)		
		σ=1.7	σ=2.7	σ=3.7	σ=1.7	σ=2.7	σ=3.7
GDP	MY	−0.40	−0.38	−0.36	−0.92	−0.89	−0.83
	IM	−0.40	−0.39	−0.36	−0.92	−0.89	−0.83
	PE	−0.29	−0.27	−0.25	−0.71	−0.68	−0.63
Employment	MY	−0.34	−0.37	−0.39	−0.79	−0.86	−0.90
	IM	−0.34	−0.37	−0.39	−0.79	−0.86	−0.89
	PE	−0.25	−0.27	−0.28	−0.61	−0.66	−0.69
Water use	MY	−0.57	−0.60	−0.60	−1.33	−1.39	−1.40
	IM	−0.57	−0.60	−0.61	−1.33	−1.38	−1.39
	PE	−0.44	−0.46	−0.46	−1.09	−1.13	−1.14

**Note**: MY = Myopic; IM = Imperfect foresight; PE = Perfect foresight; GDP = gross domestic product; σ = Armington trade elasticity.

**Table A2:** Impacts on GDP during temporal water scarcity events for Scenarios 1 and 2 with varying openness of the economy. The results give the impacts for selected time periods. DEMACRO-ESS simulation results with different investment behaviour model specifications. Values in percentage changes from base year.

		Scenario 1			Scenario 2		
		5	8	10	5	8	10
σ=1.7	MY	−0.16	−0.03	−0.02	−0.16	−0.24	−0.07
	IM	−0.16	−0.06	−0.03	−0.16	−0.27	−0.12
	PE	−0.17	−0.01	−0.01	−0.18	−0.24	−0.03
σ=2.7	MY	−0.14	−0.04	−0.02	−0.14	−0.22	−0.07
	IM	−0.14	−0.06	−0.03	−0.14	−0.24	−0.11
	PE	−0.16	−0.01	−0.01	−0.17	−0.22	−0.03
σ=3.7	MY	−0.13	−0.03	−0.02	−0.13	−0.20	−0.06
	IM	−0.13	−0.05	−0.03	−0.13	−0.22	−0.10
	PE	−0.15	−0.01	0.00	−0.16	−0.21	−0.02

**Note**: MY = Myopic; IM = Imperfect foresight; PE = Perfect foresight; σ = Armington trade elasticity.

**Table A3:** Cumulative percentage change impacts across all simulation periods of different investment behaviour model specifications on sector effects of GDP, employment, and water use during a temporal water scarcity event. DEMACRO-ESS simulation results.

	GDP			Employment			Water use		
	MY	IM	PE	MY	IM	PE	MY	IM	PE
Agriculture, forestry & fishing	−14.3	−14.3	−13.0	−7.4	−7.4	−6.7	−15.7	−15.7	−14.2
Mining & quarrying	−0.2	−0.2	−0.1	−0.1	−0.1	−0.1	−0.2	−0.2	−0.1
Crude petroleum, natural gas, ores, & coal	−0.5	−0.5	−0.4	−0.4	−0.4	−0.3	−0.6	−0.6	−0.4
Other minining & mining services	−0.5	−0.5	−0.4	−0.4	−0.4	−0.3	−0.5	−0.5	−0.4
Food (& tobacco)	−2.2	−2.2	−1.9	−2.1	−2.1	−1.9	−2.5	−2.5	−2.0
Drink	−1.4	−1.5	−1.2	−1.4	−1.4	−1.1	−1.5	−1.5	−1.2
Textile, leather, & Wood	−0.6	−0.6	−0.5	−0.5	−0.5	−0.4	−0.9	−0.9	−0.8
Paper & printing	−0.3	−0.3	−0.2	−0.1	−0.2	−0.1	−0.4	−0.4	−0.3
Coke & refined petroleum products	−0.5	−0.5	−0.3	−0.3	−0.3	−0.2	−0.6	−0.6	−0.5
Chemicals & Pharmaceuticals	−1.1	−1.1	−1.1	−0.3	−0.3	−0.3	−1.6	−1.6	−1.5
Rubber, cement, & Glass	−0.2	−0.2	−0.2	−0.2	−0.2	−0.2	−0.3	−0.3	−0.2
Iron, steel & metal	0.0	0.0	0.0	0.1	0.1	0.0	−0.1	−0.1	−0.1
Electrical manufacturing	0.1	0.1	0.1	0.1	0.1	0.1	0.0	0.0	0.0
Manufacture of motor vehicles	0.1	0.1	0.1	0.2	0.2	0.1	0.1	0.1	0.0
Transport equipment (incl Repair)	0.0	0.0	0.0	0.1	0.1	0.0	−0.1	−0.1	0.0
Electricity, transmission & distribution	−2.0	−2.0	−1.7	−1.0	−1.0	−0.8	−2.2	−2.2	−1.9
Gas & distribution of gaseous fuels	−0.6	−0.6	−0.4	−0.6	−0.6	−0.4			
Water treatment, supply, & sewarage	−0.5	−0.5	−0.3	−0.4	−0.4	−0.3	−0.6	−0.6	−0.3
Water management & remediation	−0.5	−0.5	−0.3	−0.4	−0.4	−0.2			
Construction	−0.3	−0.3	−0.1	−0.3	−0.3	−0.1	−0.4	−0.4	−0.1
Wholesale & retail trade	−0.4	−0.4	−0.3	−0.3	−0.3	−0.3	−0.5	−0.5	−0.3
Land transport	−0.2	−0.2	−0.1	−0.2	−0.2	−0.1			
Other transport	0.0	0.0	0.0	0.0	0.0	0.1			
Transport support	−0.2	−0.3	−0.2	−0.2	−0.2	−0.1			
Accommodation & food service activities	−0.2	−0.2	0.0	−0.1	−0.1	0.0	−0.2	−0.2	0.0
Communication	−0.1	−0.1	0.0	−0.1	−0.1	0.0			
Services	−0.3	−0.3	−0.1	−0.2	−0.2	−0.1	−0.3	−0.3	−0.1
Education health & defence	0.0	0.0	0.0	0.0	0.0	0.0			
Recreational	−0.2	−0.2	0.0	−0.1	−0.1	0.0			
Other private services	−0.2	−0.2	0.0	−0.1	−0.1	0.0			

**Note:** MY = Myopic; IM = Imperfect foresight; PE = Perfect foresight; GDP = gross domestic product.

**Table A4:** Cumulative percentage change impacts across all simulation periods of different investment behaviour model specifications on sector effects of GDP, employment, and water use during a temporal water scarcity svent. DEMACRO-ESS simulation results.

	GDP			Employment			Water use		
	MY	IM	PE	MY	IM	PE	MY	IM	PE
Agriculture, forestry & fishing	−32.7	−32.5	−30.2	−17.0	−16.9	−15.7	−35.8	−35.6	−33.1
Mining & quarrying	−0.4	−0.4	−0.3	−0.3	−0.3	−0.2	−0.6	−0.6	−0.4
Crude Petroleum, natural gas, ores, & coal	−1.1	−1.1	−0.9	−0.9	−0.9	−0.8	−1.3	−1.3	−1.0
Other minining & mining services	−1.1	−1.1	−0.9	−0.9	−0.9	−0.9	−1.2	−1.2	−1.0
Food (& Tobacco)	−5.2	−5.1	−4.5	−4.9	−4.9	−4.4	−5.8	−5.8	−5.0
Drink	−3.3	−3.3	−2.8	−3.1	−3.1	−2.7	−3.5	−3.5	−2.9
Textile, leather, & Wood	−1.4	−1.4	−1.3	−1.1	−1.1	−1.1	−2.1	−2.1	−1.9
Paper & printing	−0.7	−0.7	−0.5	−0.4	−0.4	−0.2	−1.0	−1.0	−0.8
Coke & refined petroleum products	−1.1	−1.1	−0.9	−0.6	−0.6	−0.5	−1.5	−1.5	−1.2
Chemicals & pharmaceuticals	−2.6	−2.6	−2.6	−0.8	−0.8	−0.8	−3.7	−3.7	−3.6
Rubber, cement, & Glass	−0.6	−0.6	−0.6	−0.4	−0.4	−0.4	−0.8	−0.8	−0.7
Iron, steel & metal	0.0	0.0	−0.1	0.2	0.2	0.0	−0.3	−0.3	−0.3
Electrical manufacturing	0.2	0.2	0.1	0.3	0.3	0.2	0.1	0.1	0.0
Manufacture of motor vehicles	0.3	0.3	0.2	0.4	0.4	0.2	0.1	0.1	0.1
Transport equipment (incl repair)	0.0	0.0	0.0	0.1	0.1	0.1	−0.2	−0.2	−0.1
Electricity, transmission & distribution	−4.7	−4.7	−4.2	−2.3	−2.3	−2.0	−5.1	−5.1	−4.6
Gas & distribution of gaseous fuels	−1.5	−1.5	−1.1	−1.3	−1.3	−1.0			
Water treatment, supply, & sewarage	−1.2	−1.2	−0.8	−1.0	−1.0	−0.6	−1.4	−1.4	−0.9
Water management & remediation	−1.2	−1.2	−0.7	−1.0	−1.0	−0.6			
Construction	−0.7	−0.7	−0.3	−0.6	−0.6	−0.3	−0.8	−0.8	−0.3
Wholesale & retail trade	−0.9	−0.9	−0.7	−0.8	−0.8	−0.7	−1.1	−1.1	−0.8
Land transport	−0.5	−0.5	−0.4	−0.4	−0.4	−0.3			
Other transport	0.0	0.0	0.1	0.1	0.1	0.1			
Transport support	−0.6	−0.6	−0.4	−0.5	−0.5	−0.4			
Accommodation & food service activities	−0.4	−0.4	0.0	−0.3	−0.3	0.1	−0.5	−0.5	−0.1
Communication	−0.3	−0.3	0.0	−0.3	−0.3	0.0			
Services	−0.7	−0.7	−0.3	−0.5	−0.5	−0.2	−0.7	−0.7	−0.4
Education health & defence	−0.1	−0.1	0.0	0.0	0.0	0.1			
Recreational	−0.4	−0.4	0.0	−0.3	−0.3	0.1			
Other private services	−0.4	−0.4	0.0	−0.3	−0.3	0.1			

**Note:** MY = Myopic; IM = Imperfect foresight; PE = Perfect foresight; GDP = gross domestic product.

## Data Availability

Data will be made available on request

## Appendix A Sensitivity Analysis

In the base DEMARCO-ESS model used for the simulations outlined in the main body of the text, the Armington trade elasticity is set to 2.7. This parameter choice is informed by empirical research conducted by Bajzik et al. (2020), where the authors employed robust Bayesian and frequentist modelling techniques. However, due to uncertainty surrounding the precise value of this elasticity, a sensitivity analysis is conducted in this section. The purpose of this analysis is to ensure that the main results remain valid under different levels of economic openness by varying the Armington elasticity. Table A1 presents the total GDP, employment, and water use results across all time periods, using Armington elasticities (σ) of 1.7, 2.7, and 3.7.

An economy that is more open tends to have a positive effect on GDP and water usage, but a negative impact on employment. When the Armington elasticity is set at 1.7, the negative effect on GDP attributed to water scarcity is 0.4%, which decreases to 0.36% with increased economic openness. However, the negative impacts on employment increase from 0.34% to 0.39% under the same conditions.

Although not the focus of this paper, it is worth considering that GDP-intensive industries may be more influenced by trade than labour-intensive industries. Additionally, it is worth noting that higher levels of economic openness are associated with a slight decrease in water usage, which is important for environmental sustainability. However, it is important to consider a caveat to these findings. The assumption is that Germany's trade partners are operating under business-as-usual conditions, which may not be the case. Some neighbouring European trade partners may also be experiencing water scarcity at the same time as Germany. Therefore, a comprehensive multi-regional framework would be needed to analyse this aspect more thoroughly.

The impact of economic openness on firms' behaviour is not significant, but it does vary in magnitude. Both myopic and imperfect foresight yield similar results over all time periods, while firms with perfect foresight can mitigate negative impacts. Table A2 in provides more details on the GDP impacts of the water scarcity simulation at different levels of economic openness. Similar to the findings in Table A1, the level of economic openness has negligible impact on the results discussed in the main report. The only difference is the magnitude of the impact. Regardless of the level of economic openness, an economy characterised by firms with perfect foresight performs better than both the myopic and imperfect foresight scenarios in all simulations. Imperfect foresight, in particular, leads to over-investment and the most significant negative impacts over the 10-month period, as shown in Table A2.

## Appendix D Further Discussion of Sectoral Results

Fig. 2 in the main text provides a visual representation of the changes in GDP, employment, and water usage across the 25 industrial sectors included in the model, considering three different investment scenarios. Detailed results are given in Appendix B, Appendix C. The results show that most industries experience negative impacts, with agriculture being the most heavily affected, as indicated by the red shading in Fig. 2. This is not surprising, as there is a direct link between agricultural output and groundwater conditions. According to the DEMARCO-ESS database, the agriculture sector accounts for 73% of freshwater consumption. A decrease in freshwater availability negatively impacts crop growth and livestock farming, resulting in reduced output and higher prices.

According to the DEMARCO-ESS database, two other industries heavily reliant on freshwater are chemical and pharmaceutical manufacturing, and electricity generation. These industries use freshwater in various processes such as heating, cooling, and as a primary input in their products. Like agriculture, these industries are directly affected by reduced freshwater availability, as it is a crucial input in their production processes. Fig. 2 in the paper visually demonstrates a significant negative impact (indicated by orange shading) of water scarcity across chemical and pharmaceutical manufacturing and electricity generation. However, the magnitude of the impact is comparatively smaller than agriculture, as these industries depend less on freshwater inputs. They account for only 1.2% and 1.4% of total inputs, respectively, compared to 8.7% for agriculture.

It is important to note that industries that do not heavily consume freshwater can still be significantly affected by water scarcity. Fig. 2 in the paper highlights two key examples in Germany: the food and beverage industries, both experiencing significant economic impacts (indicated by orange shading). While freshwater accounts for less than 0.2% of their total input values, these industries heavily rely on inputs from agriculture. Therefore, reduced agricultural output due to water scarcity leads to increased production costs in food and beverage manufacturing. Furthermore, price increases in the agriculture sector indirectly impact other industries such as textiles, leather & wood, and wholesale & retail trade. Thus, the impacts of water scarcity extend beyond industries with high freshwater consumption.

While reduced freshwater availability has an overall negative impact on the economy, some industries may experience positive effects due to changes in consumer spending patterns, particularly among households, and competitiveness trade effects. From Fig. 2, it is observed that the main industries experiencing positive impacts are electrical manufacturing and vehicle manufacturing. These sectors use minimal freshwater directly in their production processes and are less interconnected with industries heavily reliant on freshwater, except for electricity generation. As a result, their prices decrease relative to other industries, which attracts more consumers and leads to an increase in economic output. However, it is important to note that these positive effects are outweighed in aggregate by the negative impact on agriculture and food manufacturing.

## Appendix E Supplementary materials

Image, application 1

## Footnotes

1 There is a large literature that assesses the macro-level socio-economic impacts of increased water scarcity in its various forms and using a wide array of modelling approaches. A detailed review is, for example, given in Bekchanov et al. (2017) and Wittwer (2019). Supplementary Material S1 offers an overview of the research related to the modelling approach employed in this paper.

2 Typically, the literature specifies three types of resilience: engineering, ecological, and evolutionary (this is discussed in detail in Davoudi et al. (2013); Hodgson et al. (2015) and Holling (1996), for example). The focus of this paper is on engineering resilience.

3 It is important to note that while the empirical model focuses on Germany, the qualitative findings and policy recommendations have relevance for other countries and regions as well. To accurately capture the dynamics of the economy under analysis, it is essential to appropriately calibrate and parameterise the model. This crucial step ensures that the model incorporates the distinct characteristics and complexities of the specific economy being studied. However, main qualitative results presented in this paper can be seen as indicative for other European nations and regions.

4 This represents only a subset of the modelling results. Other variables, such as the impact on households, government budgets, wage incomes, land use, and so on are also available but not discussed here.

5 This is a total reduction in GDP amounting to 0.14% of an average month.

6 Appendix A provides a detailed sensitivity analysis of key parameters used in the model.

7 Further discussion on sectoral effects can be found in Appendix D.

## References

[bib0001] Allan G., Figus G., McGregor P.G., Swales J.K. (2020). Resilience in a behavioural/Keynesian regional model. Environ. Plann. A: Econ. Space.

[bib0002] Armington P.S. (1969). A theory of demand for products distinguished by place of production. Staff Papers.

[bib0003] Bajzik J., Havranek T., Irsova Z., Schwarz J. (2020). Estimating the Armington elasticity: the importance of study design and publication bias. J. Int. Econ..

[bib0004] Bekchanov M., Sood A., Pinto A., Jeuland M. (2017). Systematic review of water-economy modeling applications. J. Water. Resour. Plan. Manage.

[bib0005] Blanchflower D.G., Oswald A.J. (1995). An introduction to the wage curve. J. Econ. Perspect..

[bib0006] Calzadilla A., Rehdanz K., Roson R., Sartori M., Tol R.S.J., Alvaro C., Katrin R., Roberto R., Martina S., Richard S.J.T. (2016). The WSPC Reference on Natural Resources and Environmental Policy in the Era of Global Change.

[bib0007] Davoudi S., Brooks E., Mehmood A. (2013). Evolutionary Resilience and Strategies for Climate Adaptation. Plann. Pract. Res..

[bib0008] Dixon P.B., Jorgenson D. (2012).

[bib0009] EEA (2021). https://www.eea.europa.eu/ds_resolveuid/89f3788b2db14fc5a765fdaee007f7dc.

[bib0010] Emonts-Holley T., Ross A., Swales J. (2014). A social accounting matrix for Scotland. Fraser Allander Econ. Comment..

[bib0011] EuropeanCommission. (2021). *Economic data related to the implementation of the WFD and the FD and the financing of measures: final report*. Publications Office. 10.2779/163850.

[bib0012] Glaser R., Kahle M. (2020). Reconstructions of droughts in Germany since 1500–combining hermeneutic information and instrumental records in historical and modern perspectives. Climate Past.

[bib0013] Hanna R., Gross R. (2021). How do energy systems model and scenario studies explicitly represent socio-economic, political and technological disruption and discontinuity? Implications for policy and practitioners. Ener. Pol..

[bib0014] Hayashi F. (1982). Tobin's marginal q and average q: a neoclassical interpretation. Econometrica.

[bib0015] Hodgson D., McDonald J.L., Hosken D.J. (2015). What do you mean, ‘resilient’?. Trends Ecol. Evol. (Amst.).

[bib0016] Holling C.S. (1996). Engineering resilience versus ecological resilience. Engineer. Ecolog. Constraints.

[bib0017] Ionita M., Dima M., Nagavciuc V., Scholz P., Lohmann G. (2021). Past megadroughts in central Europe were longer, more severe and less warm than modern droughts. Commun. Earth. Environ..

[bib0018] IPCC. (2023). *Climate Change 2023: Synthesis Report*. Assessment Report of the Intergovernmental Panel on Climate Change. 10.59327/IPCC/AR6-9789291691647.

[bib0019] Kummu M., Guillaume J.H.A., de Moel H., Eisner S., Flörke M., Porkka M., Siebert S., Veldkamp T.I.E., Ward P.J. (2016). The world's road to water scarcity: shortage and stress in the 20th century and pathways towards sustainability. Sci. Rep..

[bib0020] Lecca, P., McGregor, P.G., & Swales, J.K. (2013). Forward-looking and myopic regional Computable General Equilibrium models: how significant is the distinction? *Econ. Modell.*, 31, 160–176. 10.1016/j.econmod.2012.11.010.

[bib0021] Longhi S., Nijkamp P., Poot J. (2006). Spatial heterogeneity and the wage curve revisited. J. Reg. Sci..

[bib0022] McCollum D.L., Gambhir A., Rogelj J., Wilson C. (2020). Energy modellers should explore extremes more systematically in scenarios. Nat. Energy.

[bib0023] McGregor P.G., Ross A.G., Swales J.K. (2021). How fiscal policies affect energy systems: the importance of an ‘environmental social wage. Reg. Stud..

[bib0024] Mućk J. (2017).

[bib0025] Petrovic D., Fersch B., Kunstmann H. (2022). Droughts in Germany: performance of regional climate models in reproducing observed characteristics. Nat. Hazards Earth Sys. Sci..

[bib0026] Rakovec O., Samaniego L., Hari V., Markonis Y., Moravec V., Thober S., Hanel M., Kumar R. (2022). The 2018–2020 multi-year drought sets a new benchmark in Europe. Earths. Fut..

[bib0027] Rodina L. (2019). Defining “water resilience”: debates, concepts, approaches, and gaps. WIREs. Water..

[bib0028] Ross A.G., Connolly K., Rhoden I., Vögele S. (2023). Resource-use intensity and the labour market: more for less?. Environ. Impact. Assess. Rev..

[bib32] Ross A.G., McGregor P.G., Swales J.K. (2024). Labour market dynamics in the era of technological advancements: The system-wide impacts of labour augmenting technological change. Technol. Soc..

[bib0029] Stadler K., Wood R., Bulavskaya T., Södersten C.-J., Simas M., Schmidt S., Usubiaga A., Acosta-Fernández J., Kuenen J., Bruckner M., Giljum S., Lutter S., Merciai S., Schmidt J.H., Theurl M.C., Plutzar C., Kastner T., Eisenmenger N., Erb K.-H., Tukker A. (2018). EXIOBASE 3: developing a time series of detailed environmentally extended multi-regional input-output tables. J. Ind. Ecol..

[bib0030] Toreti A., Belward A., Perez-Dominguez I., Naumann G., Luterbacher J., Cronie O., Seguini L., Manfron G., Lopez-Lozano R., Baruth B., van den Berg M., Dentener F., Ceglar A., Chatzopoulos T., Zampieri M. (2019). The exceptional 2018 European water seesaw calls for action on adaptation. Earths. Fut..

[bib0031] Wittwer G. (2019). Economy-wide modeling of water at regional and global scales. Springer Nat, Singapore.

