# Supplementary material for: A macro-level analysis of the socio-economic impacts of climate change driven water scarcity: Incorporating behavioural and resilience aspects
Source: Water Res X. 2024 Apr 16;23:100223. doi: 10.1016/j.wroa.2024.100223 (PMC11636926; doi:10.1016/j.wroa.2024.100223)
Supplement: Supplementary file 1 [file mmc1.docx]

## **Supplemental Material**

### **Supplementary material S1**: Background

Computable General Equilibrium (CGE) models are widely used to assess the economic and environmental impacts of climate change and to develop strategies for mitigation (Babatune et al, 2017; Bardazzi & Bosello, 2021). The problem of water scarcity has led to an increasing number of studies that utilise CGE models to analyse this issue. Table S1 provides a selection of these studies, highlighting various aspects related to the structure of the model (specifically the production function) and its investment foresight. Each row in the table represents an individual study. The "Production Function" column indicates whether the study explicitly includes land and/or water as factors of production in the CGE model. The "Investment Foresight" column shows the foresight model specification, with "MY" denoting Myopic foresight, "IM" representing Imperfect foresight, and "PE" corresponding to Perfect foresight. The DEMACRO-ESS model employed in the current analysis is also listed in Table S1 in the first data row to explicitly highlight its characteristics.

**Table S1**: Selection of literature that utilises Computable General Equilibrium models to study water (scarcity).

|  | Production Function | | Investment Foresight | | |
| --- | --- | --- | --- | --- | --- |
|  | Land | Water | MY | IM | PE |
| DEMACRO-ESS | X | X | X | X | X |
| Bizdazzi (2022) | X | X | X | - | - |
| Briland et al. (2023) | - | X | X | - | - |
| Briritella et al. (2008) | X | - | X | - | - |
| Calzadilla et al. (2010) | X | - | X | - | - |
| Diao & Roe (2003) | X | X | - | - | X |
| Goodman (2000) | X | X | - | - | X |
| Koopman et al. (2015) | X | - | X | - | - |
| Li et al. (2015) | - | X | X | - | - |
| Lincandeo et al. (2015) | X | X | X | - | - |
| Liu et al. (2016) | X | X | X | - | - |
| Nechifor & Winning (2017) | X | - | X | - | - |
| Nechifor & Winning (2018) | X | - | X | - | - |
| Qureshi et al. (2012) | - | X | X | - | - |
| Schuenemann & Hess (2022) | X | - | X | - | - |
| Taheripour et al. (2015) | X | X | X | - | - |
| van Heerden et al. (2008) | - | X | X | - | - |

**Note:** MY = Myopic; IM = Imperfect foresight; PE = Perfect foresight; GDP = gross domestic product.

In recent years, there has been a notable increase in the literature that incorporates both land and water into CGE models. These studies recognise the significance of considering both factors of production to capture the complexities and interactions associated with water (scarcity). While some analysis solely focuses on land separation, particularly within the agricultural sector and how drought affects land productivity, the DEMACRO-ESS model employed in this paper follows the prevailing trend in the literature by including both land and water in the model.

Many studies in this field utilise a myopic model specification, which can be justified due to the limitations of perfect foresight models in assessing unpredictable and discontinuous (extreme) events. However, perfect foresight models are valuable despite their limitations, especially since policymakers aim to encourage firms to plan for the long term. Furthermore, these models show that it sometimes beneficial to take actions now that only yield benefits in the long run, even if they might not be chosen in the short term. With perfect foresight, it is possible to avoid stranded investments resulting from decisions made under imperfect information (myopic planning). This is widely recognised in the modelling of the energy transition, where a combination of investment foresight approaches is used (Hanna & Gross, 2021; McCollum et al. 2020). However, this practice has not yet spilled over to research specifically focused on water (scarcity).

The DEMACRO-ESS model utilised in this study is noteworthy for its incorporation of different investment foresight behaviours. This represents the first attempt to explore the effects of different foresight specifications in the context of water scarcity. Additionally, the model explicitly includes both land and water as factors of production, allowing for a more comprehensive understanding of the complexities and interactions associated with water scarcity. This enhances the novelty and relevance of the study in addressing this critical issue.

**References for supplementary material S1:**

Babatunde, K., Begum., & Said, F. (2017). Application of computable general equilibrium (CGE) to climate change mitigation policy: A systematic review. Renewable and Sustainable Energy Reviews, 78, 61-71. <https://doi.org/10.1016/j.rser.2017.04.064>

Bardazzi, E. (2022). Macroeconomic, Food and Energy Security Implications of Water Dependency under a Changing Climate: A Computable General Equilibrium Assessment. Environmental Sciences Proceedings, 15(1). <https://doi.org/10.3390/environsciproc2022015001>

Bardazzi, E., & Bosello, F. (2021). Critical reflections on Water-Energy-Food Nexus in Computable General Equilibrium models: A systematic literature review. Environmental Modelling & Software, 145, 105201. <https://doi.org/10.1016/j.envsoft.2021.105201>

Berrittella, M., Hoekstra, A., Rehanz., Roson, R., & Tol, R. (2008). The economic impact of restricted water supply: A computable general equilibrium analysis. Water Research, 41(8), 1799-1813. <https://doi.org/10.1016/j.watres.2007.01.010>

Briand, A., Reynaud, A., Viroleau, F., Markantonis, V., & Branciforti, G. (2023). Environmental Modelling & Assessment, 28, 259-272. <https://doi.org/10.1007/s10666-023-09883-4>

Calzdilla, A., Rehandz, K., & Tol, R. (2010). The economic impact of more sustainable water use in agriculture:A computable general equilibrium analysis. Journal of Hydrology, 384, 292-302. <https://doi.org/10.1016/j.jhydrol.2009.12.012>

Diao, X., & Roe, T. (2003). Can a water market avert the “double-whammy” of trade reform and lead to a “win–win” outcome?. Journal of Environmental Economics and Management, 45(3), 708-723. <https://doi.org/10.1016/S0095-0696(02)00019-0>

Goodman, D. J. (2000). More reservoirs or transfers? A computable general equilibrium analysis of projected water shortages in the Arkansas River Basin. Journal of Agricultural and Resource Economics, 698-713.

Hanna, R., & Gross, R. (2021). How do energy systems model and scenario studies explicitly represent socio-economic, political and technological disruption and discontinuity? Implications for policy and practitioners. Energy Policy, 149, 111984. <https://doi.org/10.1016/j.enpol.2020.111984>

Koopman, J., Kuik, O., Tol, R., & Brouwer, R. (2015). Water scarcity from climate change and adaption response in an international river basin context. Climate Change Economics, 6(1), 1550004. <https://doi.org/10.1142/S2010007815500049>

Li, N., Wang, X., Shi., & Yang. (2015). Economic Impacts of Total Water Use Control in the Heihe River Basin in Northwestern China—An Integrated CGE-BEM Modeling Approach. Sustainability, 7(3), 3460-3478. <https://doi.org/10.3390/su7033460>

Lincandeo, F., Flores, F., & Feijoo. (2023). Assessing the impacts of economy-wide emissions policies in the water, energy, and land systems considering water scarcity scenarios. Applied Energy, 342, 121115. <https://doi.org/10.1016/j.apenergy.2023.121115>

Liu, J., Hertel, T., & Taheripour, F. (2016). Analyzing future water scarcity in Computable General Equilibrium models. Water Economic and Policy, 2(4), 1650006. <https://doi.org/10.1142/S2382624X16500065>

McCollum, D. L., Gambhir, A., Rogelj, J., & Wilson, C. (2020). Energy modellers should explore extremes more systematically in scenarios. Nature Energy, 5(2), 104-107. <https://doi.org/10.1038/s41560-020-0555-3>

Nechifor, V., & Winning. (2017). Projecting irrigation water requirements across multiple socio-economic development futures – A global CGE assessment. Water Resources and Economics, 20, 16-60. <https://doi.org/10.3390/w10101442>

Nechifor, V., & Winning. (2018). Global Economic and Food Security Impacts of Demand-Driven Water Scarcity—Alternative Water Management Options for a Thirsty World. Water, 10(10), 1442. <https://doi.org/10.3390/w10101442>

Qureshi, M., Proctor, W., Young., & Wittwer. (2012). The Economic Impact of Increased WaterDemand in Australia: A Computable General Equilibrium Analysis. Economic papers, 31(1), 87-102. <https://doi.org/10.1111/j.1759-3441.2011.00160.x>

Schuenemann, F., & Hess, S. (2022). Livestock support and water depletion in Turkey. Water Resources Research, 59(1). <https://doi.org/10.1029/2020WR028860>

Taheripour, F., Hertel, T., Gopalakrishnan, B., Sahin, K., & Escurra, J. (2015). Agricultural production, irrigation, climate change, and water scarcity in Indi. Agricultural and Applied Economics Association. <https://ideas.repec.org/p/ags/aaea15/205591.html>

van Heerden., Blignaut, J., & Horridge. (2008). Integrated water and economic modelling of the impacts of water market instruments on the South African economy. Ecological Economics, 66, 105-116. <https://doi.org/10.1016/j.ecolecon.2007.11.011>
